# Renal association commentary on the KDIGO (2017) clinical practice guideline update for the diagnosis, evaluation, prevention, and treatment of CKD-MBD

**DOI:** 10.1186/s12882-018-1037-8

**Published:** 2018-09-20

**Authors:** James O. Burton, David J. Goldsmith, Nicki Ruddock, Rukshana Shroff, Mandy Wan

**Affiliations:** 1 College of Life Sciences, University of Leicester, Leicester, UK; 2 University Hospitals of Leicester NHS Trust, Leicester, UK; 3 Guy’s and St. Thomas’ NHS Trust, London, UK; 4 Great Ormond Street Hospital for Children and University College London, London, UK; 5 Institute of Pharmaceutical Science, King’s College, London, UK

## Abstract

This report comments on the relevance and utility of the recently published (2017) KDIGO Clinical Practice Guideline Update for the diagnosis, evaluation, prevention and treatment of mineral bone disease in patients with chronic kidney disease (CKD-MBD) with respect to UK clinical practice. This document replaces all previously published Renal Association guidelines on the topic.

## Introduction

This report comments on the relevance and utility of the recently published (2017) KDIGO Clinical Practice Guideline Update for the diagnosis, evaluation, prevention and treatment of mineral bone disease in patients with chronic kidney disease (CKD-MBD) [1] with respect to UK clinical practice. This document replaces all previously published Renal Association guidelines on the topic.

In each case, we have included the guideline from the original 2009 KDIGO Report [2], followed by the updated recommendation or suggestion from the 2017 Update and a summary of the rationale behind each change. This is, of course, covered more comprehensively in the original documents.

Where UK guidance also exists from the National Institute of Health and Care Excellence (NICE) concerning individual recommendations, we have highlighted key similarities and differences before making a summary recommendation of how the Guideline Update may influence UK clinical practice.

The most significant change in the update is a move away from treating to specific targets towards a more pragmatic and personalised approach to management. It is worth noting that of the 21 updated recommendations / suggestions reviewed here, 8 of them (38%) remain ‘ungraded’ and all of the others are graded Level 2 which, according to the KDIGO nomenclature and description for rating guideline recommendations, means that ‘Different choices will be appropriate for different patients. Each patient needs help to arrive at a management decision consistent with her or his vaand preferences’ and reflects the relative lack of evidence on which clinical practice is based in this area. As a result, management of CKD-MBD in patients should be individualised, multi-professional and often pragmatic in its approach.

This represents a significant shift in the way that data are reported and interpreted by both health care professionals and patients here in the UK. As mentioned, the KDIGO working group highlighted the relative paucity of high-quality data from clinical trials that has underpinned these changes and recommend a number of priority research areas in their document. This reinforces the importance of supporting, and recruiting to, new and ongoing clinical trials wherever and whenever practically possible.

## Summary of recommendations

**Note: Guideline numbers refer to the 2017 KDIGO update**

### Chapter 3.2: Diagnosis of CKD-MBD: bone

#### Guideline 3.2.1

In patients with CKD-MBD, we suggest DXA scanning as an aid to management only if it will impact upon treatment decisions. However, we feel that the best practice includes multi-professional decision-making between local rheumatological / osteoporosis expertise and those teams looking after CKD G3a to G5D patients.

#### Guideline 3.2.2

In patients with all stages of CKD, the utility of biomarkers to predict underlying bone histology is as yet unproven; we suggest that a bone biopsy may be clinically appropriate should it lead to changes in treatment. However, implementation of this ungraded guidance may be limited dependent upon local expertise.

### Chapter 4.1: Treatment of CKD-MBD targeted at lowering high serum phosphate and maintaining serum calcium

#### Guidelines 4.1.1–4.1.2

We suggest that the treatment of CKD-MBD in all stages of CKD should be based on serial measurements (trends) of phosphate, calcium and PTH and individualised to patients.

In patients with CKD 3a-5D, a pragmatic and individualised approach to phosphate reduction towards the normal range is appropriate in the UK.

#### Guideline 4.1.3

In patients with CKD G3a-G5D, we suggest avoidance of hypercalcaemia, maintaining serum calcium below the upper limit of the reference range for the laboratory used. Hypocalcaemia should be managed in an individualised way as over-correction may also lead to patient harm.

#### Guideline 4.1.4

In patients with CKD G5D, we suggest using a dialysate calcium concentration between 1.25 and 1.5 mmol/l but, as stated in the original KDIGO document, flexibility should be maintained with dialysate calcium concentrations to meet specific, individual patient requirements.

#### Guideline 4.1.5

We suggest that phosphate binders should not be used *pre-emptively* in CKD G3a-G5D patients but reserved for those with progressively rising or persistently elevated serum phosphate. Phosphate lowering requires a multi-professional approach to therapy.

#### Guideline 4.1.6

In patients with CKD stages G3a-5D, we suggest limiting the use of calcium-based phosphate binders. Whilst calcium-based phosphate binders still have a role in the management of hyperphosphataemia in adults with CKD, their place as first line agents in the majority can no longer be recommended, especially as generic, lower cost alternatives for non-calcium containing binders are becoming more widely available.

#### Guideline 4.1.8

We suggest that limiting phosphate intake in patients with CKD G3a-5D remains integral in the management of hyperphosphataemia. With more information available about phosphate in food and its bioavailability, advice should be evidence-based, personalised and ideally delivered by Specialist Renal Dietitians.

### Chapter 4.2: Treatment of abnormal PTH levels in CKD-MBD

#### Guideline 4.2.1

We suggest that CKD G3a-G5 patients with progressively rising or persistently elevated PTH levels should be evaluated for modifiable factors, which now includes high phosphate intake and vitamin D deficiency; treatment decisions should not be based on a single elevated value.

#### Guideline 4.2.2

The updated guideline no longer recommends routine use of calcitriol or its analogs in non-dialysis CKD patients. We suggest that clinicians should reserve their use for severe and progressive SHPT, starting at low dose and avoiding hypercalcaemia.

#### Guideline 4.2.4

We suggest that calcimimetics, calcitriol and vitamin D analogues are all acceptable therapies for CKD G5D patients requiring PTH lowering therapy. Individual treatment choice should continue to be guided by considerations of concomitant therapies as well as calcium and phosphate levels.

For those patients with severe hyperparathyroidism that fail to respond to medical or pharmacological therapy, we suggest parathyroidectomy.

### Chapter 4.3: Treatment of bone with bisphosphonates, other osteoporosis medications, and growth hormone

#### Guideline 4.3

In patients with CKD G3a–G5D and evidence of MBD, we suggest that treatment choices take specific side effects into account and the risk of their administration must be weighed against the accuracy of the diagnosis of the underlying bone phenotype.

### Chapter 5: Evaluation and treatment of kidney transplant bone disease

#### Guideline 5.5

As with CKD G3a-G5D, we suggest that DXA may be a useful tool to assess fracture risk in kidney transplant recipients (G5T). Unfortunately, even when in possession of that knowledge, there is a paucity of evidence regarding interventions that reduce future fractures in transplanted patients.

#### Guideline 5.6

This group suggests considering vitamin D, calcitriol/alfacalcidol, and/or antiresorptive agents in the first year post transplant in patients with a reduced BMD and eGFR > 30 ml/min/1.73m^2^. However, as there remains no consensus on the optimal treatment strategy for mineral metabolism abnormalities, initiation of antiresorptive agents is likely to remain guided by local practice in this population.

### Paediatric specific recommendations

We suggest a calcium-based phosphate binder as the first-line phosphate reduction therapy in children and either combining with, or switching to, sevelamer if a series of serum calcium measurements shows a trend towards the age-adjusted upper limit of normal.

## Summary of audit measures

1. Percentage of adult CKD G5D patients with serum Ca above the normal reference range
2. Percentage of children with CKD G5D serum calcium below the age-appropriate normal range
3. Percentage of children with CKD G5D starting therapy with a non-calcium based phosphate binder

## Rationale

### Chapter 3.2: Diagnosis of CKD-MBD: bone

#### 2009 KDIGO guideline

3.2.1: In patients with CKD stages 3–5D, it is reasonable to perform a bone biopsy in various settings, including, but not limited to: unexplained fractures, persistent bone pain, unexplained hypercalcemia, unexplained hypophosphatemia, possible aluminium toxicity, and prior to therapy with bisphosphonates in patients with CKD–MBD (not graded).

#### 2017 KDIGO update

3.2.1: In patients with CKD G3a–G5D with evidence of CKD-MBD and/or risk factors for osteoporosis, we suggest BMD testing to assess fracture risk if results will impact treatment decisions (2B).

### Rationale

It is now well established that patients with CKD G3a–G5D have increased fracture rates compared with the general population. In addition, it is clear that incident hip fractures are associated with substantial morbidity and mortality, again, greater than that seen in age-matched members of the general population.

At the time of the 2009 KDIGO CKD-MBD guideline, publications addressing the ability of dual-energy X-ray absorptiometry (DXA) measures of bone mineral density (BMD) to estimate fracture risk in CKD were limited to cross-sectional studies comparing BMD in CKD patients with and without a prevalent fracture. The results were variable across studies and across skeletal sites. In light of that, and the inability of DXA to indicate the histological type of bone disease, the 2009 KDIGO Guideline recommended that BMD testing not be performed routinely in patients with CKD G3a to G5D with CKD-MBD. Furthermore, the lack of therapeutic clinical trials in patients with low BMD and CKD also limited the enthusiasm for measuring BMD in the first place.

The current evidence-based review identified 4 prospective cohort studies of DXA BMD and incident fractures in adults with CKD G3a to G5D. These studies demonstrated that DXA BMD predicted fractures across the spectrum from CKD G3a to G5D [3–6].

As an example of this new evidence, Naylor et al. [4] assessed the ability of the Fracture Risk Assessment Tool (FRAX) to predict a major osteoporotic fracture in 2107 adults greater than 40 years of age in the Canadian Multicenter Osteoporosis Study, including 320 with an eGFR < 60 ml/min/1.73m^2^. Of these, 72% and 24% had CKD G3a and G3b, respectively. FRAX with BMD, FRAX without BMD, and the femoral neck T-score all predicted fractures (AUC: 0.65 to 0.71); the AUC was highest for femoral neck T-score with inclusion of fall history. Importantly, the AUCs did not differ between those with and without CKD.

This is an important change between the 2009 and 2017 guidance. It begs many questions in implementation however. For example, what exactly are “risk factors for osteoporosis” in CKD patients, when CKD itself is clearly such a risk (as is age, itself tightly coupled to CKD prevalence). And, how often should these scans be repeated, and, in whom? At what DXA MBD thresholds should treatment commence? And, with what? It is also surprising to see that the FRAX score is not mentioned more prominently in the recommendations.

#### RA recommendation

In patients with CKD-MBD, we suggest DXA scanning as an aid to management only if it will impact upon treatment decisions. However, we feel that the best practice includes multi-professional decision-making between local rheumatological / osteoporosis expertise and those teams looking after CKD G3a to G5D patients.

#### 2009 KDIGO guideline

3.2.2: In patients with CKD stages 3–5D with evidence of CKD–MBD, we suggest that BMD testing not be performed routinely, because BMD does not predict fracture risk as it does in the general population, and BMD does not predict the type of renal osteodystrophy (2B).

#### 2017 KDIGO update

3.2.2: In patients with CKD G3a–G5D, it is reasonable to perform a bone biopsy if knowledge of the type of renal osteodystrophy will impact treatment decisions (Not Graded).

### Rationale

Renal osteodystrophy is defined as abnormal bone histology and is but one component of the bone abnormalities of CKD-MBD [7]. Bone biopsy is the gold standard for the diagnosis and classification for renal osteodystrophy. As detailed in the 2009 KDIGO Guideline, DXA BMD does not distinguish among types of renal osteodystrophy, and the diagnostic utility of biochemical markers is extremely limited by poor sensitivity and specificity. Differences in PTH assays (e.g. intact vs. whole PTH) and their referent ranges have contributed to differences across studies. Unfortunately, cross-sectional studies have provided conflicting information on the use of biomarkers to predict underlying bone histology. This is not so very surprising given the short half-lives of most of the circulating biomarkers, and the long (3–6 months) bone remodelling (turnover) cycle [8].

KDIGO recently led an international consortium to conduct a cross-sectional retrospective diagnostic study of biomarkers (all run in a single laboratory) and bone biopsies in 492 dialysis patients [9]. The objective was to determine the predictive value of PTH (determined by both intact PTH [iPTH] and whole PTH assays), bone-specific alkaline phosphatase (bALP), and amino-terminal propeptide of type 1 procollagen (P1NP) as markers of bone turnover. Although iPTH, whole PTH, and bALP levels were associated with bone turnover, no biomarker singly or in combination was sufficiently robust to diagnose low, normal, and high bone turnover in an individual patient. The conclusion was in support of the 2009 KDIGO Guideline to use trends in PTH rather than absolute “target” values when making decisions as to whether to start or stop treatments to lower PTH.

Although bone biopsy is an under-utilised diagnostic tool, it is inappropriate to perform them without local infrastructure to handle specimens and read the histomorphometry of paediatric and adult bone.

#### RA recommendation

In patients with all stages of CKD, the utility of biomarkers to predict underlying bone histology is as yet unproven; we suggest that a bone biopsy may be clinically appropriate should it lead to changes in treatment. However, implementation of this ungraded guidance may be limited dependent upon local expertise.

### Chapter 4.1: Treatment of CKD-MBD targeted at lowering high serum phosphate and maintaining serum calcium

#### 2009 KDIGO guideline

4.1.1: In patients with CKD stages 3–5, we suggest maintaining serum phosphorus in the normal range (2C). In patients with CKD stage 5D, we suggest lowering elevated phosphorus levels toward the normal range (2C).

#### 2017 KDIGO update

4.1.1: In patients with CKD G3a-G5D, treatments of CKD-MBD should be based on serial assessments of phosphate, calcium, and PTH levels, considered together (Not graded).

4.1.2: In patients with CKD G3a-G5D, we suggest lowering elevated phosphate levels toward the normal range (2C).

### Rationale for section 4.1.1

This revised KDIGO 2017 guidance highlights the need to ensure all variables are considered together with acknowledgement of trends, rather than specific values and is a new recommendation for KDIGO. Previous RA guidance (2015) similarly recommended making decisions based on the entire available dataset [10].

Targets set previously for individual parameters have been derived from observational data showing those levels associated with lowest risk of mortality / morbidity. Phosphate, calcium and PTH each have levels which may be associated with poorer outcomes however treatment strategies to address one of these often affects the others therefore any potential benefit that may be seen could be offset by a change in the other variable.

The combinations of different levels and their effect on outcome has been explored to highlight the complexity of the treatment of CKD-MBD [11].

### Rationale for section 4.1.2

KDIGO 2009 suggested that in CKD G3a-G5, efforts should be made to *maintain* serum phosphate in the normal range compared to actively *lowering* levels towards normal in those on dialysis G5D; the update unifies the recommendation across groups.

Despite good evidence supporting the association of higher phosphate levels with mortality across all CKD groups, there is still a lack of evidence supporting the benefits of treatment.

Previous KDIGO targets recommended that for CKD G3a-G5, phosphate levels should be *maintained* in the normal range. In practice, for many, this translated into *aiming* for levels within the normal range i.e. address hyperphosphataemia with diet and binders – there is a subtle difference.

The new recommendation has arisen as a consequence of new data in people with CKD G3a-G5 not on dialysis. A study looking at the effect of binders (calcium acetate, lanthanum carbonate and sevelamer carbonate) vs. placebo in people with eGFR 25-40 ml/min and normal/near normal serum phosphate levels demonstrated a reduction of serum phosphate from mean 4.2 mg/dl to 3.9 mg/dl (1.34 to 1.26 mmol/l) in those treated with phosphate binders compared to unchanged levels in the placebo group [12]. This was supported by reduction in phosphate excretion. Benefits were also seen in PTH levels which remained stable in the phosphate binder group (more so in the calcium acetate group) compared to an increase in levels seen in the placebo group. However, the same study demonstrated increases in calcification scores in the active treatment (phosphate binder) group than with placebo – it was most pronounced in the calcium acetate group but was still present in those taking non-calcium containing binders. It should be noted that those with a zero-calcification score at baseline, regardless of randomisation, remained unchanged as has been seen in previous studies.

Calcium carbonate was not included in this study so the effect of an even higher elemental calcium load is unknown – mean dose of calcium acetate in this study was 5.9 g (1500 mg elemental calcium) – approximately six Phosex or Renacet tablets; six Calcichew or Adcal tablets, perceived as equivalent phosphate binding capacity, would contribute 3000 / 3600 mg elemental calcium.

This study led the KDIGO group to exercise caution in promoting preventative phosphate lowering interventions in those with normal / near normal phosphate levels.

An additional mineral balance study in pre-dialysis patients (mean eGFR 36 ml/min) using calcium carbonate demonstrated no effect on phosphate balance but a positive calcium balance which may be undesirable [13].

It is worth noting that both previous UK Renal Association (2015) and NICE (2013) guidance make recommendations for specific targets of 0.9–1.5 mmol/l for CKD 3–5 and 1.1–1.7 mmol/l for CKD 5D [14]. The review group feels however that a more individualised approach is now needed, taking into account baseline phosphate, patients’ tolerance and preference for different binders, and affordability.

#### RA recommendation

We suggest that the treatment of CKD-MBD in all stages of CKD should be based on serial measurements (trends) of phosphate, calcium and PTH and individualised to patients.

In patients with CKD G3a-G5D, a pragmatic and individualised approach to phosphate reduction towards the normal range is appropriate in the UK.

#### 2009 KDIGO guideline

4.1.2: In patients with CKD stages 3–5D, we suggest maintaining serum calcium in the normal range (2D).

#### 2017 KDIGO update

4.1.3: In adult patients with CKD G3a–G5D, we suggest avoiding hypercalcemia (2C).

### Rationale

Epidemiological evidence linking higher calcium concentrations to increased mortality and non-fatal cardiovascular events in adults with CKD has accumulated since the KDIGO guideline on CKD-MBD from 2009. On this basis the grade of this guideline has changed from 2D to 2C. This is still a relatively weak evidentiary base, due to the lack of prospective studies targeting higher calcium concentrations with the aim to improve clinical outcomes. Although the original guideline suggested maintaining serum calcium in the normal range, correction of hypocalcemia carries a risk of inducing a positive calcium balance, and persistently low calcium levels in subjects treated with cinacalcet in the EVOLVE study was not harmful [15]. Therefore, the KDIGO committee considered it reasonable to consider low serum calcium within an individualised perspective, refraining from a generalised suggestion as in the previous of the KDIGO guideline to maintain calcium in the normal range for all CKD patients as mild and asymptomatic hypocalcemia (e.g., in the context of calcimimetic treatment) can be tolerated in order to avoid inappropriate calcium loading in adults.

The previous Renal Association guideline [10] suggested that albumin-adjusted serum calcium levels are kept within the normal reference range for the laboratory used and ideally maintained between 2.2 and 2.5 mmol/l, with avoidance of hypercalcaemic episodes (2D).

#### RA recommendation

In patients with CKD G3a-G5D, we suggest avoidance of hypercalcaemia, maintaining serum calcium below the upper limit of the reference range for the laboratory used. Hypocalcaemia should be managed in an individualised way as over-correction may also lead to patient harm.

#### 2009 KDIGO guideline

4.1.3: In patients with CKD G5D, we suggest using a dialysate calcium concentration between 1.25 and 1.50 mmol/l (2.5 and 3.0 mEq/l) (2D).

#### 2017 KDIGO update

4.1.4: In patients with CKD G5D, we suggest using a dialysate calcium concentration between 1.25 and 1.5 mmol/l (2.5 and 3.0 mEq/l) (2C).

### Rationale

The strength of this guideline recommendation has been upgraded from a 2D to a 2C based on two randomised controlled trials published after the original document in 2009.

The first by Spasovski et al. examined the effects of 2 different dialysate calcium concentrations in patients with adynamic bone disease; a lower dialysate calcium (1.25 mmol) was found to improve bone and mineral parameters compared with the higher concentration of 1.75 mmol [16]. The second by Ok et al. demonstrated that lowering dialysate calcium levels slowed the progression of coronary arterial calcification and improved biopsy-proven bone turnover (low bone turnover decreased from 85.0 to 41.8%) in their studied cohort of patients on HD [17]. Neither study had a comparator group using a 1.5 mmol/l calcium concentration leaving open the possibility that lower levels of dialysate calcium (> 1.25 mmol/l but < 1.75 mmol/l) would be equally beneficial.

It is worth noting here the observations of a retrospective cohort study by Brunelli et al. published in 2015 that raised some safety concerns with the default use of lower dialysate calcium concentrations (< 1.25 mmol/l) which associated with heart failure events and hypotension [18], underpinning the original 2009 KDIGO Guidelines which pointed out that a low dialysate calcium concentration may predispose to cardiac arrhythmias and intradialytic hypotension. Similarly, with higher dialysate calcium concentrations of 1.75 mmol/l there is an increased risk for all-cause mortality and cardiovascular or infection-related hospitalisation in incident HD patients [19] making their routine use in clinical practice equally unwise.

#### RA recommendation

In patients with CKD G5D, we suggest using a dialysate calcium concentration between 1.25 and 1.5 mmol/l but, as stated in the original KDIGO document, flexibility should be maintained with dialysate calcium concentrations to meet specific, individual patient requirements.

#### 2009 KDIGO guideline

4.1.4: In patients with CKD stages 3–5 (2D) and 5D (2B), we suggest using phosphate-binding agents in the treatment of hyperphosphatemia. It is reasonable that the choice of phosphate binder takes into account CKD stage, the presence of other components of CKD–MBD, concomitant therapies, and side-effect profile (not graded).

#### 2017 KDIGO update

4.1.5: In patients with CKD G3a–G5D, decisions about phosphate-lowering treatment should be based on progressively or persistently elevated serum phosphate (Not Graded).

### Rationale

A significant proportion of the rationale regarding this updated recommendation was based on two studies that were designed to investigate the effect of phosphate reduction on both FGF-23 and (soluble) Klotho levels in normophosphataemic CKD G3b-G4 patients. The results of both of these trials indicated that phosphate binding therapy should not be initiated as a preventative measure due to potential increases in calcium balance [13] and arterial calcification [12]. Consequently, the decision to treat should be based only on progressively or persistently elevated serum phosphate levels.

The other change to this recommendation was the use of the broader term “phosphate-lowering therapies” instead of phosphate-binding agents. This was included to recognise that calcium-free binders may also possess a potential for harm (e.g., due to side effects such as GI distress and binding of essential nutrients) and that all possible approaches (i.e., binders, diet, and dialysis) can be effective at controlling serum phosphate concentration.

#### RA recommendation

We suggest that phosphate binders should not be used *pre-emptively* in CKD G3a-G5D patients but reserved for those with progressively rising or persistently elevated serum phosphate. Phosphate lowering requires a multi-professional approach to therapy.

#### 2009 KDIGO guideline

4.1.5: In patients with CKD stages 3–5D and hyperphosphatemia, we recommend restricting the dose of calcium-based phosphate binders and/or the dose of calcitriol or vitamin D analog in the presence of persistent or recurrent hypercalcemia (1B).

In patients with CKD stages 3–5D and hyperphosphatemia, we suggest restricting the dose of calcium-based phosphate binders in the presence of arterial calcification (2C) and/or adynamic bone disease (2C) and/or if serum PTH levels are persistently low (2C).

#### 2017 KDIGO update

4.1.6: In adult patients with CKD G3a–G5D receiving phosphate-lowering treatment, we suggest restricting the dose of calcium-based phosphate binders (2B).

### Rationale

Calcium binders have historically been an appealing first choice; indeed, they were the main beneficiary of the flight from aluminium-based binders. Part of their popularity also arose because they also address the secondary hypocalcaemia that can be seen with hyperphosphataemia in patients with chronic kidney disease. However, overuse of calcium binders typically leads to hypercalcaemia and accelerated vascular calcification and these are the main concerns with using calcium-containing phosphate binders, particularly when they are combined with vitamin D therapy. The Kidney Disease Outcomes Quality Initiative Guidelines suggested that doses should not exceed 1500 mg/day of elemental calcium [20] based on evidence that regularly exceeding this threshold can induce a positive calcium balance (excess body stores of calcium leading to soft-tissue and vessel calcification) in chronic kidney disease [13]. However, this recommendation has never been tested in a clinical trial setting, and so there is little hard evidence to support this recommendation in clinical practice.

The updated KDIGO guideline also makes a case for restricting calcium-based phosphate binders in pre-dialysis CKD and dialysis patients based on 2 randomised trials from the *Di Iorio* group and a meta-analyses of phosphate binder trials. The INDEPENDENT trial randomised 466 incident haemodialysis patients to sevelamer or a calcium-based binder and showed that at 36 months follow-up there was a significantly lower cardiovascular mortality and death from cardiac arrhythmias in the sevelamer treated group (*p* < 0.001 for both outcomes) [21]. The same group have also shown that in 212 patients with CKD 3–4 there was a lower all-cause mortality in patients treated with sevelamer compared to calcium carbonate (*p* < 0.05) at 36 months follow-up [22].

Two meta-analyses of phosphate binder trials in CKD have shown a higher mortality in patients taking calcium-based binders compared to non-calcium based binders. Jamal et al.*..* examined data from 11 RCTs and found that patients taking sevelamer had a 22% lower mortality (RR 0.78 95% CI 0.61–0.98) compared to those taking calcium-based binders [23]. A more recent meta-analysis by Patel et al that looked at 25 studies showed that patients on sevelamer had 46% lower mortality (RR 0.54 95% CI 0.32–0.93) compared to those on calcium-based binders [24]. Of note, more recent meta-analyses may be confounded with the potential effect of widespread use of cinacalcet.

The main advantage of calcium-based binders is that they are inexpensive. The 2013 NICE guideline on hyperphosphataemia management suggests using a calcium-based binder as first line treatment in the management of hyperphosphataemia in adults with CKD 4–5 and on dialysis, and either combining with, or switching to, a non-calcium-based binder if hypercalcaemia develops, if serum parathyroid hormone levels are low or if hyperphosphataemia persists despite adherence to the maximum recommended or tolerated dose of calcium-based phosphate binder [14]. In that document, calcium acetate is the preferred calcium-based binder, sevelamer hydrochloride or lanthanum carbonate the preferred non-calcium based binders; an additional non-calcium containing binder, sucroferric oxyhydroxide was the subject of a NICE evidence summary in 2015 as it was not available when NICE published the 2013 guideline [25].

Previous UK Renal Association guidelines did not give specific recommendations on the choice of phosphate binder stating that there are insufficient data from randomised controlled trials that any specific oral phosphate binder impacts on individual patient outcome. However, the guideline did acknowledge that, given the strong association between high-dose calcium supplementation and worse outcome even in the general population, it would be reasonable to minimise calcium exposure in CKD and dialysis patients. This group continues to endorse the view that the choice of oral phosphate binder is best taken by involving both the patient and multi-disciplinary team, taking into account palatability, tolerability, efficacy, side-effect profile and affordability.

#### RA recommendation

In patients with CKD stages G3a-5D, we suggest limiting the use of calcium-based phosphate binders. Whilst calcium-based phosphate binders still have a role in the management of hyperphosphataemia in adults with CKD, their place as first line agents in the majority can no longer be recommended, especially as generic, lower cost alternatives for non-calcium containing binders are becoming more widely available.

#### 2009 KDIGO guideline

4.1.7: In patients with CKD stages 3–5D, we suggest limiting dietary phosphate intake in the treatment of hyperphosphatemia alone or in combination with other treatments (2D).

#### 2017 KDIGO update

4.1.8: In patients with CKD G3a–G5D, we suggest limiting dietary phosphate intake in the treatment of hyperphosphatemia alone or in combination with other treatments (2D). It is reasonable to consider phosphate source (e.g., animal, vegetable, additives) in making dietary recommendations (Not Graded).

### Rationale

Generally, it is well accepted that reducing phosphate intake, with or without the use of phosphate binders, helps to reduce phosphate load in people with CKD. Although studies to support this are limited and of poor quality, there is a growing evidence base around phosphate in food and it is relevant to refer to this when making dietary recommendations.

There are many difficulties when assessing the phosphate content of food. Food tables may under-estimate phosphate content and are updated infrequently so their validity may be questionable; the phosphate content of many processed foods (i.e. not fresh ingredients) often varies between brands depending on the additives used in their production. Where additives are used, it is necessary to screen ingredients lists to identify the presence of phosphate which may be identified by an ‘E’ number or by ‘Phos’ in amongst a list of chemical names; there is often no indication of quantity added. In addition, bioavailability of phosphate varies depending on the food source and whether the phosphate is organic or inorganic.

Lack of clarity around phosphate in food and its bioavailability can make dietary counselling difficult and complicated, contributing to issues with compliance. Foods that have a high organic phosphate content are often high in protein too and care needs to be taken not to compromise protein intake; protein:phosphate ratio is useful to identify the better protein sources to promote.

#### RA recommendation

We suggest that limiting phosphate intake in patients with CKD G3a-5D remains integral in the management of hyperphosphataemia. With more information available about phosphate in food and its bioavailability, advice should be evidence-based, personalised and ideally delivered by Specialist Renal Dietitians.

### Chapter 4.2: Treatment of abnormal PTH levels in CKD-MBD

#### 2009 KDIGO guideline

4.2.1: In patients with CKD stages 3–5 not on dialysis, the optimal PTH level is not known. However, we suggest that patients with levels of intact PTH (iPTH) above the upper normal limit of the assay are first evaluated for hyperphosphataemia, hypocalcaemia, and vitamin D deficiency (2C).

It is reasonable to correct these abnormalities with any or all of the following: reducing dietary phosphate intake and administering phosphate binders, calcium supplements, and/or native vitamin D (not graded).

#### 2017 KDIGO update

4.2.1: In patients with CKD G3a–G5 not on dialysis, the optimal PTH level is not known. However, we suggest that patients with levels of intact PTH progressively rising or persistently above the upper normal limit for the assay be evaluated for modifiable factors, including hyperphosphataemia, hypocalcaemia, high phosphate intake, and vitamin D deficiency (2C).

### Rationale

In the interval since the 2009 KDIGO Guideline, one eligible RCT examined the impact of cholecalciferol supplementation [26] and 3 examined the impact of phosphate binders on PTH levels in the non-dialysis CKD population [27–29]. The former showed a significant reduction in PTH values with vitamin D supplementation, with no difference between the high- or low-dose groups (reinforcing the original statement from 2009 regarding correction of vitamin D deficiency; recommendation 3.1.3). Of the 3 studies investigating the effect of phosphate control on PTH levels, one reported stable PTH levels in the active therapy (phosphate binder) group with a 21% increase in PTH in the placebo group. As a result, “high phosphate intake,” was added to the list of modifiable risk factors in the updated recommendation due to the increasing recognition that excess phosphate intake does not always result in hyperphosphatemia, especially in early CKD, but may promote SHPT.

Despite these additional data, there is still an absence of RCTs that define an optimal PTH level for patients with CKD G3a to G5, or clinical endpoints of hospitalisation, fracture, or mortality. Modest increases in PTH may actually represent an appropriate adaptive response to declining kidney function, due to its phosphaturic effects and increasing bone resistance to PTH [30], hence the optimal PTH level remains unknown.

#### RA recommendation

We suggest that CKD G3a-G5 patients with progressively rising or persistently elevated PTH levels should be evaluated for modifiable factors, which now includes high phosphate intake and vitamin D deficiency; treatment decisions should not be based on a single elevated value.

#### 2009 KDIGO guideline

4.2.2: In patients with CKD stages 3–5 not on dialysis, in whom serum PTH is progressively rising and remains persistently above the upper limit of normal for the assay despite correction of modifiable factors, we suggest treatment with calcitriol or vitamin D analogs (2C).

#### 2017 KDIGO update

4.2.2: In adult patients with CKD G3a–G5 not on dialysis, we suggest that calcitriol and vitamin D analogs not be routinely used (2C). It is reasonable to reserve the use of calcitriol and vitamin D analogs for patients with CKD G4–G5 with severe and progressive hyperparathyroidism (Not Graded).

### Rationale

Since the KDIGO CKD-MBD Guidelines were developed in 2009 two RCTs have demonstrated increased hypercalcemia without any clinically relevant benefit in PTH lowering compared to earlier studies included in the 2009 KDIGO Guidelines. Two randomised trials, PRIMO and OPERA, that examined the effect of a vitamin D analogue (Paricalcitol) vs. placebo in CKD patients with left ventricular hypertrophy, did not show a difference in left ventricular mass index or diastolic dysfunction between groups after almost 12-months of follow-up [31, 32]. The PRIMO study used higher doses of Paricalcitol at 2 μg/day while the OPERA study used 1 μg daily, and PTH levels were significantly lower in the Paricalcitol treated groups in both studies. Both studies showed that episodes of hypercalcaemia were more frequent in the Paricalcitol group compared with the placebo group. Thus, lowering serum PTH failed to demonstrate improvements in clinically relevant outcomes but showed increased risk of hypercalcemia. Accordingly, routine use of calcitriol or its analogs in CKD Stages 3a–5 is no longer recommended.

Previous UK recommendations were in concordance with the original KDIGO guideline from 2009 and suggest that treatment is considered if PTH levels are persistently above the upper reference limit for the assay in patients with CKD 3b–5 and above 9 times the upper limit of normal for patients on dialysis, suggesting that treatment is modified in order to keep PTH levels between 2 and 9 times the upper limit of normal in dialysis patients. NICE guidelines on Cinacalcet use [33] have not been modified since the publication of the EVOLVE trial [15].

#### RA recommendation

The updated guideline no longer recommends routine use of calcitriol or its analogs in non-dialysis CKD patients. We suggest that clinicians should reserve their use for severe and progressive SHPT, starting at low dose and avoiding hypercalcaemia.

#### 2009 KDIGO guideline

4.2.4: In patients with CKD stage 5D and elevated or rising PTH, we suggest calcitriol, or vitamin D analogs, or calcimimetics, or a combination of calcimimetics and calcitriol or vitamin D analogs be used to lower PTH (2B).

- It is reasonable that the initial drug selection for the treatment of elevated PTH be based on serum calcium and phosphorus levels and other aspects of CKD–MBD (not graded).
- It is reasonable that calcium or non-calcium-based phosphate binder dosage be adjusted so that treatments to control PTH do not compromise levels of phosphorus and calcium (not graded).
- We recommend that, in patients with hypercalcemia, calcitriol or another vitamin D sterol be reduced or stopped (1B).
- We suggest that, in patients with hyperphosphatemia, calcitriol or another vitamin D sterol be reduced or stopped (2D).
- We suggest that, in patients with hypocalcemia, calcimimetics be reduced or stopped depending on severity, concomitant medications, and clinical signs and symptoms (2D).
- We suggest that, if the intact PTH levels fall below two times the upper limit of normal for the assay, calcitriol, vitamin D analogs, and/or calcimimetics be reduced or stopped (2C).

#### 2017 KDIGO update

4.2.4: In patients with CKD G5D requiring PTH-lowering therapy, we suggest calcimimetics, calcitriol, or vitamin D analogs, or a combination of calcimimetics with calcitriol or vitamin D analogs (2B).

### Rationale

Recommendation 4.2.4 was re-evaluated largely based on publications of post hoc analyses of the EVOLVE trial which investigated the effect of cinacalcet on cardiovascular disease in patients undergoing dialysis [15]. Although EVOLVE did not meet its primary endpoint, the KDIGO Work Group acknowledges that subsequent pre-specified analyses suggest potential benefits of calcimimetics for stage 5D patients. Nevertheless, KDIGO explicitly endorses the presence of clinical equipoise in its narrative and the need for further research.

It should be noted that while calcimimetic therapy now appears first in the list of all acceptable treatment options, the KDIGO Work Group emphasises that calcimimetics, calcitriol, or vitamin D analogues are all acceptable first-line options, and there are no data to support clinical superiority of any one agent. In addition, the updated recommendation has removed specific statements for dosage adjustment of phosphate binder, calcitriol or other vitamin D analogues to manage levels of phosphate, PTH and calcium. The recommendation is that individual treatment choice should continue to be guided by considerations of concomitant therapies as well as calcium and phosphate levels. As such, the principal underlying this recommendation remains appropriate for UK practice.

The group also notes that the 2013 UK NICE guidance [14], as well as the previous technology appraisal guidance from 2007 (reviewed in 2013) [33], both of which provide more specific guidance in recommending the use of calcimimetics in the treatment of refractory SHPT in patients with end-stage renal disease. With respect to the latter, this group agreed that there are many instances when the risks of surgery are considered to outweigh the benefits, which are not simply limited to peri-operative risk and may include number of other patient related biopsychosocial factors. Whilst parathyroidectomy remains an important and very effective treatment option in the management of SHPT, as with other sections within this commentary, we believe that decisions around medical and surgical options for the treatment of SHPT should be individualised and holistic. As such, we endorse the KDIGO recommendation 4.2.5 from 2009, unchanged in 2017 suggesting parathyroidectomy for patients with CKD G3a–G5D with severe hyperparathyroidism who fail to respond to medical or pharmacological therapy.

#### RA recommendation

We suggest that calcimimetics, calcitriol and vitamin D analogues are all acceptable therapies for CKD G5D patients requiring PTH lowering therapy. Individual treatment choice should continue to be guided by considerations of concomitant therapies as well as calcium and phosphate levels.

For those patients with severe hyperparathyroidism that fail to respond to medical or pharmacological therapy, we suggest parathyroidectomy.

### Chapter 4.3: Treatment of bone with bisphosphonates, other osteoporosis medications, and growth hormone

#### 2009 KDIGO guideline

4.3: In patients with CKD stage 3 with biochemical abnormalities of CKD–MBD and low BMD and/or fragility fractures, we suggest that treatment choices take into account the magnitude and reversibility of the biochemical abnormalities and the progression of CKD, with consideration of a bone biopsy (2D).

#### 2017 KDIGO update

4.3: In patients with CKD G3a–G5D with biochemical abnormalities of CKD-MBD and low BMD and/or fragility fractures, we suggest that treatment choices take into account the magnitude and reversibility of the biochemical abnormalities and the progression of CKD, with consideration of a bone biopsy (2D).

### Rationale

Recommendation 4.3.3 maintains the same language but has been broadened from CKD G3a-G3b to CKD G3a-G5D in light of the revised bone biopsy recommendation (recommendation 3.2.2). Although this largely unchanged recommendation continues to emphasise the lack of definitive clinical trial evidence on the efficacy of antiresorptive medications in patients with CKD-MBD, the growing clinical experience with these agents in patients with CKD would appear to have addressed, at least in part, some of the concerns raised in the 2009 KDIGO Guideline.

The updated guideline recommendation remains broad in its treatment approach and does not support prioritising one agent over another. Treatment choices for patients with CKD at risk for fracture should consider treatment of underlying biochemical abnormalities, in addition to treatment with an antiresorptive medication. Clinicians are also asked to consider whether the type of renal osteodystrophy diagnosed by bone biopsy will inform treatment decisions, as well as to take into account the course of kidney function decline. The narrative to this recommendation further implies the need for individualised treatment strategy.

The suggested approach, perhaps more pragmatic given available evidence, is applicable in the UK. However, there may be some ambiguity facing implementation of the guideline update in clinical practice because of both the inherent challenges in using biomarkers to predict underlying bone histology, and the present practical constraints in performing bone biopsies in the UK. These deficiencies are likely to impede on the selection of an appropriate pharmacological treatment for this population. The Renal Association also notes that antiresportive therapies are not licensed in children and most are not authorised for those with eGFR < 30 ml/min/m^2^.

#### RA recommendation

In patients with CKD G3a–G5D and evidence of MBD, we suggest that treatment choices take specific side effects into account and the risk of their administration must be weighed against the accuracy of the diagnosis of the underlying bone phenotype.

### Chapter 5: Evaluation and treatment of kidney transplant bone disease

#### 2009 KDIGO guideline

5.5: In patients with an estimated glomerular filtration rate greater than approximately 30 ml/min/1.73m^2^, we suggest measuring BMD in the first 3 months after kidney transplant if they receive corticosteroids, or have risk factors for osteoporosis as in the general population (2D).

#### 2017 KDIGO update

5.5: In patients with CKD G1T–G5T with risk factors for osteoporosis, we suggest that BMD testing be used to assess fracture risk if results will alter therapy (2C).

### Rationale

The 2009 KDIGO CKD-MBD Guideline recommended BMD testing in the first 3 months following transplantation in patients with an eGFR > 30 ml/min/1.73m^2^ if they receive corticosteroids or have risk factors for osteoporosis but recommended that DXA BMD not be performed in those with CKD G4T to G5T.

As detailed above in the new aforementioned Recommendation 3.2.1, there is growing evidence that DXA BMD predicts fractures across the spectrum of CKD severity, including 4 prospective cohort studies in patients with CKD G3a to G5D [3–6]. To date, there are no prospective studies addressing the ability of DXA to predict fractures in transplant recipients. However, a retrospective cohort study conducted in 238 kidney transplant recipients with CKD G1T to G5T examined the associations of DXA BMD with fracture events [34]. One hundred sixty-one Lumbar spine and total-hip BMD results were expressed as T-scores and categorised as normal, osteopaenic or osteoporotic A total of 46 incident fractures were recorded in 53 patients. In a multivariate Cox analysis of DXA BMD results in the total hip, osteopaenia (HR: 2.7, 95% CI: 1.6–4.6) and osteoporosis (HR: 3.5, 95% CI: 1.8–6.4) were associated with significantly increased risk of fracture compared with normal BMD, independent of age, sex, and diabetes. Multivariate models were not provided for the lumbar spine BMD T-score results; however, unadjusted analyses suggested that spine BMD provided less fracture prediction compared with total hip BMD. Although this DXA study in kidney transplant recipients was not eligible for the evidence-based review due to its retrospective design, the Work Group concluded that the findings were consistent with the other studies in CKD G3a to G5D as described earlier.

#### RA recommendation

As with CKD G3a-G5D, we suggest that DXA may be a useful tool to assess fracture risk in kidney transplant recipients (G5T). Unfortunately, even when in possession of that knowledge, there is a paucity of evidence regarding interventions that reduce future fractures in transplanted patients.

#### 2009 KDIGO guideline

In patients in the first 12 months after kidney transplant with an estimated glomerular filtration rate greater than approximately 30 ml/min/1.73m^2^ and low BMD, we suggest that treatment with vitamin D, calcitriol/alfacalcidol, or bisphosphonates be considered (2D).

- We suggest that treatment choices be influenced by the presence of CKD–MBD, as indicated by abnormal levels of calcium, phosphorus, PTH, alkaline phosphatases, and 25(OH)D (2C)
- It is reasonable to consider a bone biopsy to guide treatment, specifically before the use of bisphosphonates due to the high incidence of adynamic bone disease (not graded)

There are insufficient data to guide treatment after the first 12 months.

#### 2017 KDIGO update

5.6: In patients in the first 12 months after kidney transplant with an estimated glomerular filtration rate greater than approximately 30 ml/min/1.73m^2^ and low BMD, we suggest that treatment with vitamin D, calcitriol/alfacalcidol, and/or antiresorptive agents be considered (2D).

- We suggest that treatment choices be influenced by the presence of CKD-MBD, as indicated by abnormal levels of calcium, phosphate, PTH, alkaline phosphatases, and 25(OH)D (2C)
- It is reasonable to consider a bone biopsy to guide treatment (Not Graded)

There are insufficient data to guide treatment after the first 12 months.

### Rationale

Consistent with the new pragmatic bone biopsy recommendation, recommendation 5.6 has been revised to indicate that the KDIGO Work Group no longer suggests that a bone biopsy be performed prior to initiation of antiresorptive therapy in patients with CKD at risk of fracture including the kidney transplant population.

This updated recommendation maintains the prior broad suggestion to consider available therapies to prioritise treatment of underlying abnormalities in calcium, phosphate, PTH and vitamin D levels. While the agents (vitamin D, calcitriol/alfacalcidol, antiresorptive agents) are identical to the ones previously listed, the less favourable data on cinacalcet and denosumab are highlighted in the narrative of the recommendation, specifically that cincacalcet did not improve mineralisation, whereas denosumab did but was associated with more UTIs (although the reason for the UTIs was not clear). The guideline update also asks the clinician to consider bone biopsy if results will assist therapeutic decisions. This is in keeping with the recommendation for the non-transplant population, and thus faces the same challenges in implementation as described under guideline recommendation 4.3.3. This guideline update also reiterates the 2009 recommendation in that there are insufficient data to guide treatment after the first 12 months after kidney transplantation.

#### RA recommendation

This group suggests considering vitamin D, calcitriol/alfacalcidol, and/or antiresorptive agents In the first year post transplant in patients with a reduced BMD and eGFR > 30 ml/min/1.73m^2^. However, as there remains no consensus on the optimal treatment strategy for mineral metabolism abnormalities, initiation of antiresorptive agents is likely to remain guided by local practice in this population.

### The 2017 guideline update sections that relate specifically to children

#### 2009 KDIGO guideline

4.1.3: In children with CKD G3a–G5D, we suggest maintaining serum calcium in the age-appropriate normal range (2C).

4.1.6: In children with CKD G3a–G5D, it is reasonable to base the choice of phosphate-lowering treatment on serum calcium levels (Not Graded).

4.2.2: In children, calcitriol and vitamin D analogs may be considered to maintain serum calcium levels in the age-appropriate normal range (Not Graded).

#### 2017 KDIGO update

4.1.3: In children with CKD G3a–G5D, we suggest maintaining serum calcium in the age-appropriate normal range (2C).

4.1.6: In children with CKD G3a–G5D, it is reasonable to base the choice of phosphate-lowering treatment on serum calcium levels (Not Graded).

4.2.2: In children, calcitriol and vitamin D analogs may be considered to maintain serum calcium levels in the age-appropriate normal range (Not Graded).

### Rationale

In the updated KDIGO guidelines the work group recognise the higher calcium requirements of the growing skeleton and recommend that when considering phosphate binder treatment or use of vitamin D analogues, the serum calcium levels are maintained in the age-appropriate normal range. This is reflected in the updated guidelines 4.1.3, 4.1.6 and 4.2.2.

Childhood and adolescence are critical periods for bone mass accrual: in healthy children the calcium content of the skeleton increases from ~ 25 g at birth to ~ 1000 g in adults, and ~ 25% of total skeletal mass is laid down during the 2-year interval of peak height velocity [35]. The updated evidence review identified a prospective cohort study in 170 children and adolescents with CKD stages 2–5 and dialysis that showed lower serum calcium levels were independently associated with lower tibial cortical volumetric bone mineral density (BMD) Z-scores [35]. Over a one-year follow-up (*n* = 89 children) a change in the cortical BMD Z-score directly correlated with baseline calcium (*p* = 0.008) and change in calcium (*p* = 0.002) levels, particularly in growing children. 6.5% of children sustained a fracture during the one-year follow-up, and lower cortical BMD predicted future fractures: the hazard ratio for fracture was 1.75 (95% confidence interval 1.15–2.67; *p* = 0.009) per standard deviation decrease in baseline BMD [36].

Only two randomised controlled trials have examined phosphate lowering therapy in children with CKD or on dialysis; due to the small patient numbers and short follow-up both studies were excluded from the evidence review for the guideline update. The first RCT examined biochemical end-points only and showed equivalent phosphate control with calcium acetate and sevelamer hydrochloride in an 8-week cross-over trial [37]. In the second, 29 children were randomised to different combinations of phosphate binders and vitamin D analogues: bone biopsies suggested that the sevelamer group had reduced bone formation at 8-month follow-up, but numbers were too small for comparison [38]. A recent prospective cohort study in 537 children with pre-dialysis CKD reported that phosphate binder treatment (calcium based in 82%) was associated with decreased risk of incident fractures (HR 0.37, 95% CI 0.15–0.941), independent of age, sex, eGFR, and PTH levels [39]. Although this study did not meet the criteria for recommendation for treatment, it highlights the need for additional studies in children.

Given the association of high PTH levels with reduced bone mineralisation and vascular calcification, children are likely to need calcitriol or other active vitamin D analog therapy. A recent Cochrane review has examined vitamin D therapy for bone disease in children with CKD stages 2–5 and on dialysis [40]. Bone disease, as assessed by changes in PTH levels, was improved by all vitamin D preparations regardless of preparation or route or frequency of administration. High PTH levels were independently associated with reduced tibial cortical BMD Z-scores and are associated with coronary artery calcification in children on dialysis [41]. The NICE guideline on the management of hyperphosphataemia in adults with CKD [14] offers similar advice on phosphate binder treatment in children.

#### RA recommendation

We suggest a calcium-based phosphate binder as the first-line phosphate reduction therapy in children and either combining with, or switching to, sevelamer if a series of serum calcium measurements shows a trend towards the age-adjusted upper limit of normal.
